# A new *S*-type upper bound for the largest singular value of nonnegative rectangular tensors

**DOI:** 10.1186/s13660-017-1382-3

**Published:** 2017-05-09

**Authors:** Jianxing Zhao, Caili Sang

**Affiliations:** College of Data Science and Information Engineering, Guizhou Minzu University, Guiyang, Guizhou 550025 P.R. China

**Keywords:** 15A18, 15A42, 15A69, nonnegative tensor, rectangular tensor, singular value

## Abstract

By breaking $N=\{1,2,\ldots,n\}$ into disjoint subsets *S* and its complement, a new *S*-type upper bound for the largest singular value of nonnegative rectangular tensors is given and proved to be better than some existing ones. Numerical examples are given to verify the theoretical results.

## Introduction

Singular value problems of rectangular tensors have become an important topic in applied mathematics and numerical multilinear algebra, and it has a wide range of practical applications, such as the strong ellipticity condition problem in solid mechanics [1, 2] and the entanglement problem in quantum physics [3, 4].

Let $\mathbb{R}$ (respectively, $\mathbb{C}$) be the real (respectively, complex) field. Assume that $p,q,m,n$ are positive integers, $m,n\geq2$, $l=p+q$, and $N=\{1,2,\ldots,n\}$. A real $(p,q)$th order $m\times n$ dimensional rectangular tensor (or simply a real rectangular tensor) $\mathcal{A}$ is defined as follows:

$$\mathcal{A}=(a_{i_{1}\cdots i_{p}j_{1}\cdots j_{q}}),\quad a_{i_{1}\cdots i_{p}j_{1}\cdots j_{q}}\in\mathbb{R}, 1\leq i_{1},\ldots,i_{p}\leq m, 1\leq j_{1},\ldots ,j_{q}\leq n. $$

A real rectangular tensor $\mathcal{A}$ is called nonnegative if $a_{i_{1}\cdots i_{p}j_{1}\cdots j_{q}}\geq0$ for $i_{k}=1,\ldots,m, k=1,\ldots, p$, and $j_{v}=1,\ldots, n,v=1,\ldots,q$.

For vectors $x=(x_{1},\ldots, x_{m})^{\textrm{T}}$, $y=(y_{1},\ldots,y_{n})^{\textrm{T}}$ and a real number *α*, let $x^{[\alpha]}=(x_{1}^{\alpha},x_{2}^{\alpha},\ldots, x_{m}^{\alpha})^{\textrm{T}}$, $y^{[\alpha]}=(y_{1}^{\alpha},y_{2}^{\alpha},\ldots,y_{n}^{\alpha})^{\textrm{T}}$, $\mathcal{A}x^{p-1}y^{q}$ be an *m* dimension real vector whose *i*th component is

$$\bigl(\mathcal{A}x^{p-1}y^{q}\bigr)_{i}=\sum _{i_{2},\ldots,i_{p}=1}^{m}\sum_{j_{1},\ldots,j_{q}=1}^{n}a_{ii_{2}\cdots i_{p}j_{1}\cdots j_{q}}x_{i_{2}} \cdots x_{i_{p}}y_{j_{1}}\cdots y_{j_{q}}, $$

and $\mathcal{A}x^{p}y^{q-1}$ be an *n* dimension real vector whose *j*th component is

$$\bigl(\mathcal{A}x^{p}y^{q-1}\bigr)_{j}=\sum _{i_{1},\ldots,i_{p}=1}^{m}\sum_{j_{2},\ldots,j_{q}=1}^{n}a_{i_{1}\cdots i_{p}jj_{2}\cdots j_{q}}x_{i_{1}} \cdots x_{i_{p}}y_{j_{2}}\cdots y_{j_{q}}. $$

If $\lambda\in\mathbb{C}$, $x\in\mathbb{C}^{m}\backslash\{0\}$, and $y\in \mathbb{C}^{n}\backslash\{0\}$ are solutions of

$$ \textstyle\begin{cases} \mathcal{A}x^{p-1}y^{q}=\lambda x^{[l-1]},\\ \mathcal{A}x^{p}y^{q-1}=\lambda y^{[l-1]}, \end{cases} $$

then we say that *λ* is a singular value of $\mathcal{A}$, *x* and *y* are a left and a right eigenvectors of $\mathcal{A}$, associated with *λ*. If $\lambda\in\mathbb{R}, x\in\mathbb{R}^{m}$, and $y\in\mathbb{R}^{n}$, then we say that *λ* is an H-singular value of $\mathcal{A}$, *x* and *y* are a left and a right H-eigenvectors of $\mathcal{A}$, associated with H-singular value *λ* [5]. Here,

$$\lambda_{0}=\max\bigl\{ |\lambda|:\lambda \text{ is a singular value of } \mathcal{A}\bigr\} $$

is called the largest singular value [6].

The definition of singular values for tensors was first introduced in [7]. Note here that when *l* is even, the definitions in [5] is the same as in [7], and when *l* is odd, the definition in [5] is slightly different from that in [7], but parallel to the definition of eigenvalues of square matrices [8]; see [5] for details.

Recently, many people focus on bounding the largest singular value for nonnegative rectangular tensors [6, 9, 10]. For convenience, we first give some notation. Given a nonempty proper subset *S* of *N*, we denote

$$\begin{aligned}& \Delta^{N}:=\bigl\{ (i_{2},\ldots, i_{p},j_{1}, \ldots,j_{q}): i_{2},\ldots, i_{p},j_{1}, \ldots ,j_{q}\in N\bigr\} , \\& \Delta^{S}:=\bigl\{ (i_{2},\ldots, i_{p},j_{1}, \ldots,j_{q}): i_{2},\ldots, i_{p},j_{1}, \ldots ,j_{q}\in S\bigr\} , \\& \Omega^{N}:=\bigl\{ (i_{1},\ldots, i_{p},j_{2}, \ldots,j_{q}): i_{1},\ldots, i_{p},j_{2}, \ldots ,j_{q}\in N\bigr\} , \\& \Omega^{S}:=\bigl\{ (i_{1},\ldots, i_{p},j_{2}, \ldots,j_{q}): i_{1},\ldots, i_{p},j_{2}, \ldots ,j_{q}\in S\bigr\} , \end{aligned}$$

and then

$$\overline{\Delta^{S}}=\Delta^{N}\backslash \Delta^{S},\qquad \overline{\Omega ^{S}}=\Omega^{N} \backslash\Omega^{S}. $$

This implies that, for a nonnegative rectangular tensor $\mathcal {A}=(a_{i_{1}\cdots i_{p}j_{1}\cdots j_{q}})$, we have, for $i,j\in S$,

$$\begin{aligned}& r_{i}(\mathcal{A})=\sum_{i_{2},\ldots,i_{p},j_{1},\ldots,j_{q}\in N\atop \delta_{ii_{2}\cdots i_{p}j_{1}\cdots j_{q}}=0}a_{ii_{2}\cdots i_{p}j_{1}\cdots j_{q}}=r_{i}^{\Delta^{S}}( \mathcal{A})+r_{i}^{\overline{\Delta^{S}}}(\mathcal{A}),\quad r_{i}^{j}( \mathcal{A})=r_{i}(\mathcal{A})-a_{ij\cdots jj\cdots j}, \\& c_{j}(\mathcal{A})=\sum_{i_{1},\ldots,i_{p},j_{2},\ldots,j_{q}\in N\atop \delta_{i_{1}\cdots i_{p}jj_{2}\cdots j_{q}}=0}a_{i_{1}\cdots i_{p}jj_{2}\cdots j_{q}}=c_{j}^{\Omega^{S}}( \mathcal{A})+c_{j}^{\overline{\Omega^{S}}}(\mathcal{A}),\quad c_{j}^{i}( \mathcal{A})=c_{j}(\mathcal{A})-a_{i\cdots iji\cdots i}, \end{aligned}$$

where

$$\delta_{i_{1}\cdots i_{p}j_{1}\cdots j_{q}}= \textstyle\begin{cases} 1,& \text{if }i_{1}=\cdots=i_{p}=j_{1}=\cdots=j_{q},\\ 0,&\text{otherwise}, \end{cases} $$

and

$$\begin{aligned}& r_{i}^{\Delta^{S}}(\mathcal{A})=\sum_{(i_{2},\ldots,i_{p},j_{1},\ldots ,j_{q})\in\Delta^{S}\atop \delta_{ii_{2}\cdots i_{p}j_{1}\cdots j_{q}}=0}a_{ii_{2}\cdots i_{p}j_{1}\cdots j_{q}},\qquad r_{i}^{\overline{\Delta ^{S}}}( \mathcal{A})=\sum_{(i_{2},\ldots,i_{p},j_{1},\ldots,j_{q})\in \overline{\Delta^{S}}}a_{ii_{2}\cdots i_{p}j_{1}\cdots j_{q}}, \\& c_{j}^{\Omega^{S}}(\mathcal{A})=\sum_{(i_{1},\ldots,i_{p},j_{2},\ldots ,j_{q})\in\Omega^{S}\atop \delta_{i_{1}\cdots i_{p}jj_{2}\cdots j_{q}}=0}a_{i_{1}\cdots i_{p}jj_{2}\cdots j_{q}},\qquad c_{j}^{\overline{\Omega ^{S}}}( \mathcal{A})=\sum_{(i_{1},\ldots,i_{p},j_{2},\ldots,j_{q})\in \overline{\Omega^{S}}}a_{i_{1}\cdots i_{p}jj_{2}\cdots j_{q}}. \end{aligned}$$

In [6], Yang and Yang gave the following bound for the largest singular value of a nonnegative rectangular tensor $\mathcal{A}$.

### Theorem 1

[6], Theorem 4

*Let* $\mathcal{A}$ *be a* $(p,q)$ *th order* $m\times n$ *dimensional nonnegative rectangular tensor*. *Then*

$$\lambda_{0}\leq\max_{1\leq i\leq m, 1\leq j\leq n}\bigl\{ R_{i}( \mathcal {A}),C_{j}(\mathcal{A})\bigr\} , $$

*where*

$$R_{i}(\mathcal{A})=\sum_{i_{2},\ldots,i_{p}=1}^{m} \sum_{j_{1},\ldots ,j_{q}=1}^{n}a_{ii_{2}\cdots i_{p}j_{1}\cdots j_{q}}, \qquad C_{j}(\mathcal{A})=\sum_{i_{1},\ldots,i_{p}=1}^{m} \sum_{j_{2},\ldots,j_{q}=1}^{n}a_{i_{1}\cdots i_{p}jj_{2}\cdots j_{q}}. $$

When $m=n$, He *et al.* [9] have given an upper bound which is lower than that in Theorem 1.

### Theorem 2

[9], Theorem 1.3

*Let* $\mathcal{A}$ *be a* $(p,q)$ *th order* $n\times n$ *dimensional nonnegative rectangular tensor*. *Then*

$$\lambda_{0}\leq\Phi(\mathcal{A})=\max\bigl\{ \Phi_{1}( \mathcal{A}),\Phi _{2}(\mathcal{A}),\Phi_{3}(\mathcal{A}), \Phi_{4}(\mathcal{A})\bigr\} , $$

*where*

$$\begin{aligned} \Phi_{1}(\mathcal{A}) =&\max_{i,j\in N,i\neq j}\frac{1}{2} \bigl\{ a_{i\cdots ii\cdots i}+a_{j\cdots jj\cdots j}+r_{i}^{j}( \mathcal{A}) \\ &{}+ \bigl[\bigl(a_{i\cdots ii\cdots i}-a_{j\cdots jj\cdots j}+r_{i}^{j}( \mathcal {A})\bigr)^{2}+4a_{ij\cdots jj\cdots j}r_{j}(\mathcal{A}) \bigr]^{\frac{1}{2}} \bigr\} , \\ \Phi_{2}(\mathcal{A}) =& \max_{i,j\in N,i\neq j}\frac{1}{2} \bigl\{ a_{i\cdots ii\cdots i}+a_{j\cdots jj\cdots j}+c_{i}^{j}( \mathcal{A}) \\ &{}+ \bigl[\bigl(a_{i\cdots ii\cdots i}-a_{j\cdots jj\cdots j}+c_{i}^{j}( \mathcal {A})\bigr)^{2}+4a_{j\cdots jij\cdots j}c_{j}(\mathcal{A}) \bigr]^{\frac{1}{2}} \bigr\} , \\ \Phi_{3}(\mathcal{A}) =& \max_{i,j\in N,i\neq j}\frac{1}{2} \bigl\{ a_{i\cdots ii\cdots i}+a_{j\cdots jj\cdots j}+r_{i}^{j}( \mathcal{A}) \\ &{}+ \bigl[\bigl(a_{i\cdots ii\cdots i}-a_{j\cdots jj\cdots j}+r_{i}^{j}( \mathcal {A})\bigr)^{2}+4a_{ij\cdots jj\cdots j}c_{j}(\mathcal{A}) \bigr]^{\frac{1}{2}} \bigr\} , \\ \Phi_{4}(\mathcal{A}) =& \max_{i,j\in N,i\neq j}\frac{1}{2} \bigl\{ a_{i\cdots ii\cdots i}+a_{j\cdots jj\cdots j}+c_{i}^{j}( \mathcal{A}) \\ &{}+ \bigl[\bigl(a_{i\cdots ii\cdots i}-a_{j\cdots jj\cdots j}+c_{i}^{j}( \mathcal {A})\bigr)^{2}+4a_{j\cdots jij\cdots j}r_{j}(\mathcal{A}) \bigr]^{\frac{1}{2}} \bigr\} . \end{aligned}$$

Similarly, under the condition of $m=n$, by breaking $N=\{1,2,\ldots,n\} $ into disjoint subsets *S* and its complement *S̄*, Zhao and Sang [10] provided an *S*-type upper bound for the largest singular value of nonnegative rectangular tensors.

### Theorem 3

[10], Theorem 2.2

*Let* $\mathcal{A}$ *be a* $(p,q)$ *th order* $n\times n$ *dimensional nonnegative rectangular tensor*, *S* *be a nonempty proper subset of* *N*, *S̄* *be the complement of* *S* *in* *N*. *Then*

$$\lambda_{0}\leq U^{S}(\mathcal{A})=\max\bigl\{ U_{1}^{S}(\mathcal{A}),U_{1}^{\bar {S}}( \mathcal{A}),U_{2}^{S}(\mathcal{A}),U_{2}^{\bar{S}}( \mathcal{A})\bigr\} , $$

*where*

$$\begin{aligned} U_{1}^{S}(\mathcal{A}) =&\max_{i\in S,j\in\bar{S}} \frac{1}{2} \bigl\{ a_{i\cdots ii\cdots i}+a_{j\cdots jj\cdots j}+r_{j}^{\overline{\Delta ^{S}}}( \mathcal{A}) \\ &{}+ \bigl[\bigl(a_{i\cdots ii\cdots i}-a_{j\cdots jj\cdots j}-r_{j}^{\overline {\Delta^{S}}}( \mathcal{A})\bigr)^{2}+4\max\bigl\{ r_{i}(\mathcal{A}),c_{i}( \mathcal{A})\bigr\} r_{j}^{\Delta^{S}}(\mathcal{A}) \bigr]^{\frac{1}{2}} \bigr\} , \\ U_{1}^{\bar{S}}(\mathcal{A}) =&\max_{i\in\bar{S},j\in S} \frac {1}{2} \bigl\{ a_{i\cdots ii\cdots i}+a_{j\cdots jj\cdots j}+r_{j}^{\overline {\Delta^{\bar{S}}}}( \mathcal{A}) \\ &{}+ \bigl[\bigl(a_{i\cdots ii\cdots i}-a_{j\cdots jj\cdots j}-r_{j}^{\overline {\Delta^{\bar{S}}}}( \mathcal{A})\bigr)^{2}+4\max\bigl\{ r_{i}(\mathcal{A}),c_{i}( \mathcal {A})\bigr\} r_{j}^{\Delta^{\bar{S}}}(\mathcal{A}) \bigr]^{\frac{1}{2}} \bigr\} , \\ U_{2}^{S}(\mathcal{A}) =&\max_{i\in S,j\in\bar{S}} \frac{1}{2} \bigl\{ a_{i\cdots ii\cdots i}+a_{j\cdots jj\cdots j}+c_{j}^{\overline{\Omega ^{S}}}( \mathcal{A}) \\ &{}+ \bigl[\bigl(a_{i\cdots ii\cdots i}-a_{j\cdots jj\cdots j}-c_{j}^{\overline {\Omega^{S}}}( \mathcal{A})\bigr)^{2}+4\max\bigl\{ r_{i}(\mathcal{A}),c_{i}( \mathcal{A})\bigr\} c_{j}^{\Omega^{S}}(\mathcal{A}) \bigr]^{\frac{1}{2}} \bigr\} , \\ U_{2}^{\bar{S}}(\mathcal{A}) =&\max_{i\in\bar{S},j\in S} \frac {1}{2} \bigl\{ a_{i\cdots ii\cdots i}+a_{j\cdots jj\cdots j}+c_{j}^{\overline {\Omega^{\bar{S}}}}( \mathcal{A}) \\ &{}+ \bigl[\bigl(a_{i\cdots ii\cdots i}-a_{j\cdots jj\cdots j}-c_{j}^{\overline {\Omega^{\bar{S}}}}( \mathcal{A})\bigr)^{2}+4\max\bigl\{ r_{i}(\mathcal{A}),c_{i}( \mathcal {A})\bigr\} c_{j}^{\Omega^{\bar{S}}}(\mathcal{A}) \bigr]^{\frac{1}{2}} \bigr\} . \end{aligned}$$

In this paper, we continue this research, and give a new *S*-type upper bound for the largest singular value of nonnegative rectangular tensors. It is proved that the new upper bound is better than those in Theorems 1-3.

## Main results

### Theorem 4

*Let* $\mathcal{A}$ *be a* $(p,q)$ *th order* $n\times n$ *dimensional nonnegative rectangular tensor*, *S* *be a nonempty proper subset of* *N*, *S̄* *be the complement of* *S* *in* *N*. *Then*

$$\lambda_{0}\leq\Psi^{S}(\mathcal{A})=\max\bigl\{ \Psi_{1}^{S}(\mathcal{A}),\Psi _{1}^{\bar{S}}( \mathcal{A}),\Psi_{2}^{S}(\mathcal{A}),\Psi_{2}^{\bar {S}}( \mathcal{A}),\Psi_{3}^{S}(\mathcal{A}),\Psi_{3}^{\bar{S}}( \mathcal {A}),\Psi_{4}^{S}(\mathcal{A}),\Psi_{4}^{\bar{S}}( \mathcal{A})\bigr\} , $$

*where*

$$\begin{aligned} \Psi_{1}^{S}(\mathcal{A}) =&\max_{i\in S,j\in\bar{S}} \frac{1}{2} \bigl\{ a_{i\cdots ii\cdots i}+a_{j\cdots jj\cdots j}+r_{i}^{\Delta^{S}}( \mathcal {A})+r_{j}^{\overline{\Delta^{S}}}(\mathcal{A}) \\ &{}+ \bigl[\bigl(a_{i\cdots ii\cdots i}-a_{j\cdots jj\cdots j}+r_{i}^{\Delta ^{S}}( \mathcal{A})-r_{j}^{\overline{\Delta^{S}}}(\mathcal {A})\bigr)^{2}+4r_{i}^{\overline{\Delta^{S}}}( \mathcal{A})r_{j}^{\Delta^{S}}(\mathcal {A}) \bigr]^{\frac{1}{2}} \bigr\} , \\ \Psi_{1}^{\bar{S}}(\mathcal{A}) =&\max_{i\in\bar{S},j\in S} \frac {1}{2} \bigl\{ a_{i\cdots ii\cdots i}+a_{j\cdots jj\cdots j}+r_{i}^{\Delta ^{\bar{S}}}( \mathcal{A})+r_{j}^{\overline{\Delta^{\bar{S}}}}(\mathcal {A}) \\ &{}+ \bigl[\bigl(a_{i\cdots ii\cdots i}-a_{j\cdots jj\cdots j}+r_{i}^{\Delta^{\bar {S}}}( \mathcal{A})-r_{j}^{\overline{\Delta^{\bar{S}}}}(\mathcal {A})\bigr)^{2}+4r_{i}^{\overline{\Delta^{\bar{S}}}}( \mathcal{A})r_{j}^{\Delta^{\bar {S}}}(\mathcal{A}) \bigr]^{\frac{1}{2}} \bigr\} , \\ \Psi_{2}^{S}(\mathcal{A}) =&\max_{i\in S,j\in\bar{S}} \frac{1}{2} \bigl\{ a_{i\cdots ii\cdots i}+a_{j\cdots jj\cdots j}+c_{i}^{\Omega ^{S}}( \mathcal{A})+c_{j}^{\overline{\Omega^{S}}}(\mathcal{A}) \\ &{}+ \bigl[\bigl(a_{i\cdots ii\cdots i}-a_{j\cdots jj\cdots j}+c_{i}^{\Omega ^{S}}( \mathcal{A})-c_{j}^{\overline{\Omega^{S}}}(\mathcal {A})\bigr)^{2}+4c_{i}^{\overline{\Omega^{S}}}( \mathcal{A})c_{j}^{\Omega^{S}}(\mathcal {A}) \bigr]^{\frac{1}{2}} \bigr\} , \\ \Psi_{2}^{\bar{S}}(\mathcal{A}) =&\max_{i\in\bar{S},j\in S} \frac {1}{2} \bigl\{ a_{i\cdots ii\cdots i}+a_{j\cdots jj\cdots j}+c_{i}^{\Omega ^{\bar{S}}}( \mathcal{A})+c_{j}^{\overline{\Omega^{\bar{S}}}}(\mathcal {A}) \\ &{}+ \bigl[\bigl(a_{i\cdots ii\cdots i}-a_{j\cdots jj\cdots j}+c_{i}^{\Omega^{\bar {S}}}( \mathcal{A})-c_{j}^{\overline{\Omega^{\bar{S}}}}(\mathcal {A})\bigr)^{2}+4c_{i}^{\overline{\Omega^{\bar{S}}}}( \mathcal{A})c_{j}^{\Omega^{\bar {S}}}(\mathcal{A}) \bigr]^{\frac{1}{2}} \bigr\} , \\ \Psi_{3}^{S}(\mathcal{A}) =&\max_{i\in S,j\in\bar{S}} \frac{1}{2} \bigl\{ a_{i\cdots ii\cdots i}+a_{j\cdots jj\cdots j}+r_{i}^{\Delta^{S}}( \mathcal {A})+c_{j}^{\overline{\Omega^{S}}}(\mathcal{A}) \\ &{}+ \bigl[\bigl(a_{i\cdots ii\cdots i}-a_{j\cdots jj\cdots j}+r_{i}^{\Delta ^{S}}( \mathcal{A})-c_{j}^{\overline{\Omega^{S}}}(\mathcal {A})\bigr)^{2}+4r_{i}^{\overline{{\Delta^{S}}}}( \mathcal{A})c_{j}^{\Omega^{S}}(\mathcal {A}) \bigr]^{\frac{1}{2}} \bigr\} , \\ \Psi_{3}^{\bar{S}}(\mathcal{A}) =&\max_{i\in\bar{S},j\in S} \frac {1}{2} \bigl\{ a_{i\cdots ii\cdots i}+a_{j\cdots jj\cdots j}+r_{j}^{\overline {\Delta^{\bar{S}}}}( \mathcal{A})+c_{i}^{\Omega^{\bar{S}}}(\mathcal{A}) \\ &{}+ \bigl[\bigl(a_{i\cdots ii\cdots i}-a_{j\cdots jj\cdots j}-r_{j}^{\overline {\Delta^{\bar{S}}}}( \mathcal{A})+c_{i}^{\Omega^{\bar{S}}}(\mathcal {A})\bigr)^{2}+4r_{j}^{\Delta^{\bar{S}}}( \mathcal{A})c_{i}^{{\overline{\Omega ^{\bar{S}}}}}(\mathcal{A}) \bigr]^{\frac{1}{2}} \bigr\} , \\ \Psi_{4}^{S}(\mathcal{A}) =&\max_{i\in S,j\in\bar{S}} \frac{1}{2} \bigl\{ a_{i\cdots ii\cdots i}+a_{j\cdots jj\cdots j}+r_{j}^{\overline{\Delta ^{S}}}( \mathcal{A})+c_{i}^{\Omega^{S}}(\mathcal{A}) \\ &{}+ \bigl[\bigl(a_{i\cdots ii\cdots i}-a_{j\cdots jj\cdots j}-r_{j}^{\overline {\Delta^{S}}}( \mathcal{A})+c_{i}^{\Omega^{S}}(\mathcal{A})\bigr)^{2}+4r_{j}^{\Delta ^{S}}( \mathcal{A})c_{i}^{{\overline{\Omega^{S}}}}(\mathcal{A}) \bigr]^{\frac {1}{2}} \bigr\} , \\ \Psi_{4}^{\bar{S}}(\mathcal{A}) =&\max_{i\in\bar{S},j\in S} \frac {1}{2} \bigl\{ a_{i\cdots ii\cdots i}+a_{j\cdots jj\cdots j}+r_{i}^{\Delta ^{\bar{S}}}( \mathcal{A})+c_{j}^{\overline{\Omega^{\bar{S}}}}(\mathcal{A}) \\ &{}+ \bigl[\bigl(a_{i\cdots ii\cdots i}-a_{j\cdots jj\cdots j}+r_{i}^{\Delta ^{\bar{S}}}( \mathcal{A})-c_{j}^{\overline{\Omega^{\bar{S}}}}(\mathcal {A})\bigr)^{2}+4r_{i}^{{\overline{\Delta^{\bar{S}}}}}( \mathcal{A})c_{j}^{\Omega ^{\bar{S}}}(\mathcal{A}) \bigr]^{\frac{1}{2}} \bigr\} . \end{aligned}$$

### Proof

Because $\lambda_{0}$ is the largest singular value of $\mathcal{A}$, from Theorem 2 in [6], there are nonnegative nonzero vectors $x=(x_{1},x_{2},\ldots,x_{n})^{\mathrm{T}}$ and $y=(y_{1},y_{2},\ldots,y_{n})^{\mathrm{T}}$, such that

1 $$\begin{aligned}& \mathcal{A}x^{p-1}y^{q}=\lambda_{0} x^{[l-1]}, \end{aligned}$$

2 $$\begin{aligned}& \mathcal{A}x^{p}y^{q-1}=\lambda_{0} y^{[l-1]}. \end{aligned}$$

Let

$$\begin{aligned}& x_{t}=\max\{x_{i}:i\in S\},\qquad x_{h}=\max \{x_{i}:i\in\bar{S}\};\\& y_{f}=\max\{y_{i}:i\in S\},\qquad y_{g}=\max\{y_{i}:i\in\bar{S}\}; \\& w_{i}=\max\{x_{i},y_{i}\}, \quad i\in N,\qquad w_{S}=\max\{w_{i}:i\in S\},\qquad w_{\bar{S}}=\max\{ w_{i}:i\in\bar{S}\}. \end{aligned}$$

Then at least one of $x_{t}$ and $x_{h}$ is nonzero, and at least one of $y_{f}$ and $y_{g}$ is nonzero. We next divide into four cases to prove.

Case I: If $w_{S}=x_{t}, w_{\bar{S}}=x_{h}$, then $x_{t}\geq y_{t}, x_{h}\geq y_{h}$.

(i) If $x_{h}\geq x_{t}$, then $x_{h}=\max\{w_{i}:i\in N\}$. From (3) of Theorem 2.2 in [10], we have

3 $$\begin{aligned} & \bigl(\lambda_{0} -a_{h\cdots hh\cdots h}-r_{h}^{\overline{\Delta^{S}}}( \mathcal {A})\bigr)x_{h}^{l-1}\leq r_{h}^{\Delta^{S}}( \mathcal{A})x_{t}^{l-1}. \end{aligned}$$

If $x_{t}=0$, by $x_{h}>0$, we have $\lambda_{0} -a_{h\cdots hh\cdots h}-r_{h}^{\overline{\Delta^{S}}}(\mathcal{A})\leq0$. Then $\lambda_{0}\leq a_{h\cdots hh\cdots h}+r_{h}^{\overline{\Delta^{S}}}(\mathcal {A})\leq\Psi_{1}^{S}(\mathcal{A})$. Otherwise, $x_{t}>0$. From (1), we have

$$\begin{aligned} (\lambda_{0} -a_{t\cdots tt\cdots t})x_{t}^{l-1} \leq& \lambda_{0} x_{t}^{l-1}-a_{t\cdots tt\cdots t}x_{t}^{p-1}y_{t}^{q} \\ =&\sum_{(i_{2},\ldots, i_{p},j_{1},\ldots, j_{q})\in\Delta^{S} \atop \delta_{ti_{2}\cdots i_{p}j_{1}\cdots j_{q}}=0}a_{ti_{2}\cdots i_{p}j_{1}\cdots j_{q}}x_{i_{2}} \cdots x_{i_{p}}y_{j_{1}}\cdots y_{j_{q}} \\ &{}+\sum_{(i_{2},\ldots, i_{p},j_{1},\ldots, j_{q})\in\overline{\Delta ^{S}}}a_{ti_{2}\cdots i_{p}j_{1}\cdots j_{q}}x_{i_{2}} \cdots x_{i_{p}}y_{j_{1}}\cdots y_{j_{q}} \\ \leq&\sum_{(i_{2},\ldots, i_{p},j_{1},\ldots, j_{q})\in\Delta^{S} \atop \delta_{ti_{2}\cdots i_{p}j_{1}\cdots j_{q}}=0}a_{ti_{2}\cdots i_{p}j_{1}\cdots j_{q}}x_{t}^{l-1} +\sum_{(i_{2},\ldots, i_{p},j_{1},\ldots, j_{q})\in\overline{\Delta^{S}}} a_{ti_{2}\cdots i_{p}j_{1}\cdots j_{q}}x_{h}^{l-1} \\ =& r_{t}^{\Delta^{S}}(\mathcal{A})x_{t}^{l-1}+r_{t}^{\overline{\Delta ^{S}}}( \mathcal{A})x_{h}^{l-1}, \end{aligned}$$

*i.e.*,

4 $$\begin{aligned} & \bigl(\lambda_{0} -a_{t\cdots tt\cdots t}-r_{t}^{\Delta^{S}}( \mathcal {A})\bigr)x_{t}^{l-1}\leq r_{t}^{\overline{\Delta^{S}}}( \mathcal{A})x_{h}^{l-1}. \end{aligned}$$

If $\lambda_{0} -a_{t\cdots tt\cdots t}-r_{t}^{\Delta^{S}}(\mathcal{A})\leq 0$, then $\lambda_{0}\leq a_{t\cdots tt\cdots t}+r_{t}^{\Delta^{S}}(\mathcal{A})\leq \Psi_{1}^{S}(\mathcal{A})$. If $\lambda_{0} -a_{t\cdots tt\cdots t}-r_{t}^{\Delta^{S}}(\mathcal{A})> 0$, multiplying (3) with (4) and noting that $x_{t}^{l-1}x_{h}^{l-1}>0$, we have

5 $$\begin{aligned} & \bigl(\lambda_{0}-a_{t\cdots tt\cdots t}-r_{t}^{\Delta^{S}}( \mathcal{A})\bigr) \bigl(\lambda _{0} -a_{h\cdots hh\cdots h}-r_{h}^{\overline{\Delta^{S}}}( \mathcal{A})\bigr) \leq r_{t}^{\overline{\Delta^{S}}}(\mathcal{A})r_{h}^{\Delta^{S}}( \mathcal{A}). \end{aligned}$$

Solving $\lambda_{0}$ in (5) gives

$$\begin{aligned} \lambda_{0} \leq& \frac{1}{2} \bigl\{ a_{t\cdots tt\cdots t}+a_{h\cdots hh\cdots h}+r_{t}^{\Delta^{S}}( \mathcal{A})+r_{h}^{\overline{\Delta ^{S}}}(\mathcal{A}) \\ &{}+ \bigl[\bigl(a_{t\cdots tt\cdots t}+r_{t}^{\Delta^{S}}( \mathcal{A})-a_{h\cdots hh\cdots h}-r_{h}^{\overline{\Delta^{S}}}(\mathcal{A}) \bigr)^{2}+4r_{t}^{\overline {\Delta^{S}}}(\mathcal{A})r_{h}^{\Delta^{S}}( \mathcal{A}) \bigr]^{\frac {1}{2}} \bigr\} \\ \leq&\max_{i\in S,j\in\bar{S}}\frac{1}{2} \bigl\{ a_{i\cdots ii\cdots i}+a_{j\cdots jj\cdots j}+r_{i}^{\Delta^{S}}( \mathcal {A})+r_{j}^{\overline{\Delta^{S}}}(\mathcal{A}) \\ &{}+ \bigl[\bigl(a_{i\cdots ii\cdots i}-a_{j\cdots jj\cdots j}+r_{i}^{\Delta ^{S}}( \mathcal{A})-r_{j}^{\overline{\Delta^{S}}}(\mathcal {A})\bigr)^{2}+4r_{i}^{\overline{\Delta^{S}}}( \mathcal{A})r_{j}^{\Delta^{S}}(\mathcal {A}) \bigr]^{\frac{1}{2}} \bigr\} \\ =&\Psi_{1}^{S}(\mathcal{A}). \end{aligned}$$

(ii) If $x_{t}\geq x_{h}$, similar to the proof of (i), we have

$$\begin{aligned} & \bigl(\lambda_{0} -a_{h\cdots hh\cdots h}-r_{h}^{\Delta^{\bar{S}}}( \mathcal {A})\bigr) \bigl(\lambda_{0} -a_{t\cdots tt\cdots t}-r_{t}^{\overline{\Delta^{\bar {S}}}}( \mathcal{A})\bigr)\leq r_{h}^{\overline{\Delta^{\bar{S}}}}(\mathcal {A})r_{t}^{\Delta^{\bar{S}}}(\mathcal{A}), \end{aligned}$$

and

$$\begin{aligned} \lambda_{0} \leq& \frac{1}{2} \bigl\{ a_{h\cdots hh\cdots h}+a_{t\cdots tt\cdots t}+r_{h}^{\Delta^{\bar{S}}}( \mathcal{A})+r_{t}^{\overline{\Delta ^{\bar{S}}}}(\mathcal{A}) \\ &{}+ \bigl[\bigl(a_{h\cdots hh\cdots h}-a_{t\cdots tt\cdots t}+r_{h}^{\Delta^{\bar {S}}}( \mathcal{A})-r_{t}^{\overline{\Delta^{\bar{S}}}}(\mathcal {A})\bigr)^{2}+4r_{h}^{\overline{\Delta^{\bar{S}}}}( \mathcal{A})r_{t}^{\Delta^{\bar {S}}}(\mathcal{A}) \bigr]^{\frac{1}{2}} \bigr\} \\ \leq&\max_{i\in\bar{S},j\in S}\frac{1}{2} \bigl\{ a_{i\cdots ii\cdots i}+a_{j\cdots jj\cdots j}+r_{i}^{\Delta^{\bar{S}}}( \mathcal {A})+r_{j}^{\overline{\Delta^{\bar{S}}}}(\mathcal{A}) \\ &{}+ \bigl[\bigl(a_{i\cdots ii\cdots i}-a_{j\cdots jj\cdots j}+r_{i}^{\Delta^{\bar {S}}}( \mathcal{A})-r_{j}^{\overline{\Delta^{\bar{S}}}}(\mathcal {A})\bigr)^{2}+4r_{i}^{\overline{\Delta^{\bar{S}}}}( \mathcal{A})r_{j}^{\Delta^{\bar {S}}}(\mathcal{A}) \bigr]^{\frac{1}{2}} \bigr\} \\ =&\Psi_{1}^{\bar{S}}(\mathcal{A}). \end{aligned}$$

Case II: Assume that $w_{S}=y_{f}, w_{\bar{S}}=y_{g}$. If $y_{g}\geq y_{f}$, similar to the proof of (i), we have

$$\begin{aligned} & \bigl(\lambda_{0} -a_{f\cdots ff\cdots f}-c_{f}^{\Omega^{S}}( \mathcal {A})\bigr) \bigl(\lambda_{0} -a_{g\cdots gg\cdots g}-c_{g}^{\overline{\Omega ^{S}}}( \mathcal{A})\bigr)\leq c_{f}^{\overline{\Omega^{S}}}(\mathcal {A})c_{g}^{\Omega^{S}}(\mathcal{A}), \end{aligned}$$

and

$$\begin{aligned} \lambda_{0} \leq& \frac{1}{2} \bigl\{ a_{f\cdots ff\cdots f}+a_{g\cdots gg\cdots g}+c_{f}^{\Omega^{S}}( \mathcal{A})+c_{g}^{\overline{\Omega ^{S}}}(\mathcal{A}) \\ &{}+ \bigl[\bigl(a_{f\cdots ff\cdots f}-a_{g\cdots gg\cdots g}+c_{f}^{\Omega ^{S}}( \mathcal{A})-c_{g}^{\overline{\Omega^{S}}}(\mathcal {A})\bigr)^{2}+4c_{f}^{\overline{\Omega^{S}}}( \mathcal{A})c_{g}^{\Omega^{S}}(\mathcal {A}) \bigr]^{\frac{1}{2}} \bigr\} \\ \leq&\max_{i\in S,j\in\bar{S}}\frac{1}{2} \bigl\{ a_{i\cdots ii\cdots i}+a_{j\cdots jj\cdots j}+c_{i}^{\Omega^{S}}( \mathcal {A})+c_{j}^{\overline{\Omega^{S}}}(\mathcal{A}) \\ &{}+ \bigl[\bigl(a_{i\cdots ii\cdots i}-a_{j\cdots jj\cdots j}+c_{i}^{\Omega ^{S}}( \mathcal{A})-c_{j}^{\overline{\Omega^{S}}}(\mathcal {A})\bigr)^{2}+4c_{i}^{\overline{\Omega^{S}}}( \mathcal{A})c_{j}^{\Omega^{S}}(\mathcal {A}) \bigr]^{\frac{1}{2}} \bigr\} \\ =&\Psi_{2}^{S}(\mathcal{A}). \end{aligned}$$

If $y_{f}\geq y_{g}$, similarly, we have

$$\begin{aligned} & \bigl(\lambda_{0} -a_{g\cdots gg\cdots g}-c_{g}^{\Omega^{\bar{S}}}( \mathcal {A})\bigr) \bigl(\lambda_{0} -a_{f\cdots ff\cdots f}-c_{f}^{\overline{\Omega^{\bar {S}}}}( \mathcal{A})\bigr)\leq c_{g}^{\overline{\Omega^{\bar{S}}}}(\mathcal {A})c_{f}^{\Omega^{\bar{S}}}(\mathcal{A}) \end{aligned}$$

and

$$\begin{aligned} \lambda_{0} \leq& \frac{1}{2} \bigl\{ a_{g\cdots gg\cdots g}+a_{f\cdots ff\cdots f}+c_{g}^{\Omega^{\bar{S}}}( \mathcal{A})+c_{f}^{\overline{\Omega ^{\bar{S}}}}(\mathcal{A}) \\ &{}+ \bigl[\bigl(a_{g\cdots g\cdots g}-a_{f\cdots ff\cdots f}+c_{g}^{\Omega^{\bar {S}}}( \mathcal{A})-c_{f}^{\overline{\Omega^{\bar{S}}}}(\mathcal {A})\bigr)^{2}+4c_{g}^{\overline{\Omega^{\bar{S}}}}( \mathcal{A})c_{f}^{\Omega^{\bar {S}}}(\mathcal{A}) \bigr]^{\frac{1}{2}} \bigr\} \\ \leq&\max_{i\in\bar{S},j\in S}\frac{1}{2} \bigl\{ a_{i\cdots ii\cdots i}+a_{j\cdots jj\cdots j}+c_{i}^{\Omega^{\bar{S}}}( \mathcal {A})+c_{j}^{\overline{\Omega^{\bar{S}}}}(\mathcal{A}) \\ &{}+ \bigl[\bigl(a_{i\cdots ii\cdots i}-a_{j\cdots jj\cdots j}+c_{i}^{\Omega^{\bar {S}}}( \mathcal{A})-c_{j}^{\overline{\Omega^{\bar{S}}}}(\mathcal {A})\bigr)^{2}+4c_{i}^{\overline{\Omega^{\bar{S}}}}( \mathcal{A})c_{j}^{\Omega^{\bar {S}}}(\mathcal{A}) \bigr]^{\frac{1}{2}} \bigr\} \\ =&\Psi_{2}^{\bar{S}}(\mathcal{A}). \end{aligned}$$

Case III: Assume that $w_{S}=x_{t}, w_{\bar{S}}=y_{g}$. If $y_{g}\geq x_{t}$, similar to the proof of (i), we have

$$\begin{aligned} & \bigl(\lambda_{0}-a_{t\cdots tt\cdots t}-r_{t}^{\Delta^{S}}( \mathcal{A})\bigr) \bigl(\lambda _{0} -a_{g\cdots gg\cdots g}-c_{g}^{\overline{\Omega^{S}}}( \mathcal{A})\bigr) \leq r_{t}^{\overline{{\Delta^{S}}}}(\mathcal{A})c_{g}^{\Omega^{S}}( \mathcal{A}) \end{aligned}$$

and

$$\begin{aligned} \lambda_{0} \leq& \frac{1}{2} \bigl\{ a_{t\cdots tt\cdots t}+a_{g\cdots gg\cdots g}+r_{t}^{\Delta^{S}}( \mathcal{A})+c_{g}^{\overline{\Omega ^{S}}}(\mathcal{A}) \\ &{}+ \bigl[\bigl(a_{t\cdots tt\cdots t}-a_{g\cdots gg\cdots g}+r_{t}^{\Delta ^{S}}( \mathcal{A})-c_{g}^{\overline{\Omega^{S}}}(\mathcal {A})\bigr)^{2}+4r_{t}^{\overline{{\Delta^{S}}}}( \mathcal{A})c_{g}^{\Omega^{S}}(\mathcal {A}) \bigr]^{\frac{1}{2}} \bigr\} \\ \leq&\max_{i\in S,j\in\bar{S}}\frac{1}{2} \bigl\{ a_{i\cdots ii\cdots i}+a_{j\cdots jj\cdots j}+r_{i}^{\Delta^{S}}( \mathcal {A})+c_{j}^{\overline{\Omega^{S}}}(\mathcal{A}) \\ &{}+ \bigl[\bigl(a_{i\cdots ii\cdots i}-a_{j\cdots jj\cdots j}+r_{i}^{\Delta ^{S}}( \mathcal{A})-c_{j}^{\overline{\Omega^{S}}}(\mathcal {A})\bigr)^{2}+4r_{i}^{\overline{{\Delta^{S}}}}( \mathcal{A})c_{j}^{\Omega^{S}}(\mathcal {A}) \bigr]^{\frac{1}{2}} \bigr\} \\ =&\Psi_{3}^{S}(\mathcal{A}). \end{aligned}$$

If $x_{t}\geq y_{g}$, similarly, we have

$$\begin{aligned} & \bigl(\lambda_{0} -a_{g\cdots gg\cdots g}-c_{g}^{\Omega^{\bar{S}}}( \mathcal {A})\bigr) \bigl(\lambda_{0} -a_{t\cdots tt\cdots t}-r_{t}^{\overline{\Delta^{\bar {S}}}}( \mathcal{A})\bigr)\leq c_{g}^{{\overline{\Omega^{\bar{S}}}}}(\mathcal {A})r_{t}^{\Delta^{\bar{S}}}(\mathcal{A}) \end{aligned}$$

and

$$\begin{aligned} \lambda_{0} \leq& \frac{1}{2} \bigl\{ a_{t\cdots tt\cdots t}+a_{g\cdots gg\cdots g}+r_{t}^{\overline{\Delta^{\bar{S}}}}( \mathcal{A})+c_{g}^{\Omega ^{\bar{S}}}(\mathcal{A}) \\ &{}+ \bigl[\bigl(a_{t\cdots tt\cdots t}-a_{g\cdots gg\cdots g}+r_{t}^{\overline {\Delta^{\bar{S}}}}( \mathcal{A})-c_{g}^{\Omega^{\bar{S}}}(\mathcal {A})\bigr)^{2}+4r_{t}^{\Delta^{\bar{S}}}( \mathcal{A})c_{g}^{{\overline{\Omega ^{\bar{S}}}}}(\mathcal{A}) \bigr]^{\frac{1}{2}} \bigr\} \\ \leq&\max_{i\in\bar{S},j\in S}\frac{1}{2} \bigl\{ a_{i\cdots ii\cdots i}+a_{j\cdots jj\cdots j}+r_{j}^{\overline{\Delta^{\bar {S}}}}( \mathcal{A})+c_{i}^{\Omega^{\bar{S}}}(\mathcal{A}) \\ &{}+ \bigl[\bigl(a_{i\cdots ii\cdots i}-a_{j\cdots jj\cdots j}-r_{j}^{\overline {\Delta^{\bar{S}}}}( \mathcal{A})+c_{i}^{\Omega^{\bar{S}}}(\mathcal {A})\bigr)^{2}+4r_{j}^{\Delta^{\bar{S}}}( \mathcal{A})c_{i}^{{\overline{\Omega ^{\bar{S}}}}}(\mathcal{A}) \bigr]^{\frac{1}{2}} \bigr\} \\ =&\Psi_{3}^{\bar{S}}(\mathcal{A}). \end{aligned}$$

Case IV: Assume that $w_{S}=y_{f}, w_{\bar{S}}=x_{h}$. If $x_{h}\geq y_{f}$, similar to the proof of (i), we have

$$\begin{aligned} & \bigl(\lambda_{0}-a_{f\cdots ff\cdots f}-c_{f}^{\Omega^{S}}( \mathcal{A})\bigr) \bigl(\lambda _{0} -a_{h\cdots hh\cdots h}-r_{h}^{\overline{\Delta^{S}}}( \mathcal{A})\bigr) \leq c_{f}^{{\overline{\Omega^{S}}}}(\mathcal{A})r_{h}^{\Delta^{S}}( \mathcal{A}) \end{aligned}$$

and

$$\begin{aligned} \lambda_{0} \leq& \frac{1}{2} \bigl\{ a_{f\cdots ff\cdots f}+a_{h\cdots hh\cdots h}+r_{h}^{\overline{\Delta^{S}}}( \mathcal{A})+c_{f}^{\Omega ^{S}}(\mathcal{A}) \\ &{}+ \bigl[\bigl(a_{f\cdots ff\cdots f}-a_{h\cdots hh\cdots h}-r_{h}^{\overline {\Delta^{S}}}( \mathcal{A})+c_{f}^{\Omega^{S}}(\mathcal{A})\bigr)^{2}+4c_{f}^{{\overline {\Omega^{S}}}}( \mathcal{A})r_{h}^{\Delta^{S}}(\mathcal{A}) \bigr]^{\frac {1}{2}} \bigr\} \\ \leq&\max_{i\in S,j\in\bar{S}}\frac{1}{2} \bigl\{ a_{i\cdots ii\cdots i}+a_{j\cdots jj\cdots j}+r_{j}^{\overline{\Delta^{S}}}( \mathcal {A})+c_{i}^{\Omega^{S}}(\mathcal{A}) \\ &{}+ \bigl[\bigl(a_{i\cdots ii\cdots i}-a_{j\cdots jj\cdots j}-r_{j}^{\overline {\Delta^{S}}}( \mathcal{A})+c_{i}^{\Omega^{S}}(\mathcal{A})\bigr)^{2}+4c_{i}^{{\overline {\Omega^{S}}}}( \mathcal{A})r_{j}^{\Delta^{S}}(\mathcal{A}) \bigr]^{\frac {1}{2}} \bigr\} \\ =&\Psi_{4}^{S}(\mathcal{A}). \end{aligned}$$

If $y_{f}\geq x_{h}$, similarly, we have

$$\begin{aligned} & \bigl(\lambda_{0} -a_{h\cdots hh\cdots h}-r_{h}^{\Delta^{\bar{S}}}( \mathcal {A})\bigr) \bigl(\lambda_{0} -a_{f\cdots ff\cdots f}-c_{f}^{\overline{\Omega^{\bar {S}}}}( \mathcal{A})\bigr)\leq r_{h}^{{\overline{\Delta^{\bar{S}}}}}(\mathcal {A})c_{f}^{\Omega^{\bar{S}}}(\mathcal{A}) \end{aligned}$$

and

$$\begin{aligned} \lambda_{0} \leq& \frac{1}{2} \bigl\{ a_{h\cdots hh\cdots h}+a_{f\cdots ff\cdots f}+r_{h}^{\Delta^{\bar{S}}}( \mathcal{A})+c_{f}^{\overline{\Omega ^{\bar{S}}}}(\mathcal{A}) \\ &{}+ \bigl[\bigl(a_{h\cdots hh\cdots h}-a_{f\cdots ff\cdots f}+r_{h}^{\Delta ^{\bar{S}}}( \mathcal{A})-c_{f}^{\overline{\Omega^{\bar{S}}}}(\mathcal {A})\bigr)^{2}+4r_{h}^{{\overline{\Delta^{\bar{S}}}}}( \mathcal{A})c_{f}^{\Omega ^{\bar{S}}}(\mathcal{A}) \bigr]^{\frac{1}{2}} \bigr\} \\ \leq&\max_{i\in\bar{S},j\in S}\frac{1}{2} \bigl\{ a_{i\cdots ii\cdots i}+a_{j\cdots jj\cdots j}+r_{i}^{\Delta^{\bar{S}}}( \mathcal {A})+c_{j}^{\overline{\Omega^{\bar{S}}}}(\mathcal{A}) \\ &{}+ \bigl[\bigl(a_{i\cdots ii\cdots i}-a_{j\cdots jj\cdots j}+r_{i}^{\Delta ^{\bar{S}}}( \mathcal{A})-c_{j}^{\overline{\Omega^{\bar{S}}}}(\mathcal {A})\bigr)^{2}+4r_{i}^{{\overline{\Delta^{\bar{S}}}}}( \mathcal{A})c_{j}^{\Omega ^{\bar{S}}}(\mathcal{A}) \bigr]^{\frac{1}{2}} \bigr\} \\ =&\Psi_{4}^{\bar{S}}(\mathcal{A}). \end{aligned}$$

The conclusion follows from Cases I, II, III and IV. □

We next give the following comparison theorem for these upper bounds in Theorems 1-4.

### Theorem 5

*Let* $\mathcal{A}$ *be a* $(p,q)$ *th order* $n\times n$ *dimensional nonnegative rectangular tensor*, *S be a nonempty proper subset of* *N*, *S̄* *be the complement of* *S* *in* *N*. *Then*

$$\Psi^{S}(\mathcal{A})\leq U^{S}(\mathcal{A})\leq\Phi( \mathcal{A})\leq\max_{i,j\in N}\bigl\{ R_{i}( \mathcal{A}),C_{j}(\mathcal{A})\bigr\} . $$

### Proof

I. By Remark 2.2 in [9], $\Phi(\mathcal{A})\leq\max_{i,j\in N}\{R_{i}(\mathcal{A}),C_{j}(\mathcal{A})\}$ holds.

II. Next, we prove $U^{S}(\mathcal{A})\leq\Phi(\mathcal{A})$. Here, we only prove $U_{1}^{S}(\mathcal{A})\leq\Phi(\mathcal{A})$. Similarly, we can prove $U_{1}^{\bar{S}}(\mathcal{A}),U_{2}^{S}(\mathcal{A}), U_{2}^{\bar{S}}(\mathcal{A})\leq\Phi(\mathcal{A})$, respectively.

(i) Suppose that

$$\begin{aligned} U_{1}^{S}(\mathcal{A}) =& \max_{i\in S,j\in\bar{S}} \frac{1}{2} \bigl\{ a_{i\cdots ii\cdots i}+a_{j\cdots jj\cdots j}+r_{j}^{\overline{\Delta ^{S}}}( \mathcal{A})\\ &{}+ \bigl[\bigl(a_{i\cdots ii\cdots i}-a_{j\cdots jj\cdots j}-r_{j}^{\overline{\Delta^{S}}}( \mathcal{A})\bigr)^{2}+4r_{i}(\mathcal {A})r_{j}^{\Delta^{S}}( \mathcal{A}) \bigr]^{\frac{1}{2}} \bigr\} . \end{aligned}$$

From the proof of Theorem 2.2 in [10], we can see that the bound $U_{1}^{S}(\mathcal{A})$ is obtained by solving $\lambda_{0}$ from

6 $$\begin{aligned} & (\lambda_{0}-a_{i\cdots ii\cdots i}) \bigl( \lambda_{0} -a_{j\cdots jj\cdots j}-r_{j}^{\overline{\Delta^{S}}}( \mathcal{A})\bigr) \leq r_{i}(\mathcal{A})r_{j}^{\Delta^{S}}( \mathcal{A}). \end{aligned}$$

From the proof of Theorem 1.3 in [9], we can see that the bound

$$\begin{aligned} \Phi_{1}(\mathcal{A}) =& \max_{i,j\in N,i\neq j}\frac{1}{2} \bigl\{ a_{i\cdots ii\cdots i}+a_{j\cdots jj\cdots j}+r_{j}^{i}( \mathcal{A})\\ &{} + \bigl[\bigl(a_{i\cdots ii\cdots i}-a_{j\cdots jj\cdots j}-r_{j}^{i}( \mathcal {A})\bigr)^{2}+4a_{ji\cdots ii\cdots i}r_{i}(\mathcal{A}) \bigr]^{\frac{1}{2}} \bigr\} \end{aligned}$$

is obtained by solving $\lambda_{0}$ from

7 $$\begin{aligned} & (\lambda_{0}-a_{i\cdots ii\cdots i}) \bigl( \lambda_{0} -a_{j\cdots jj\cdots j}-r_{j}^{i}( \mathcal{A})\bigr)\leq a_{ji\cdots ii\cdots i}r_{i}(\mathcal{A}). \end{aligned}$$

Taking $i\in S$, $j\in\bar{S}$ in (7), by the proof of Theorem 6 in [11], we know that if $\lambda_{0}$ satisfies (6), then $\lambda _{0}$ satisfies (7), which implies that

$$\begin{aligned} \Phi_{1}(\mathcal{A}) \geq&\max_{i\in S,j\in\bar{S}} \frac {1}{2} \bigl\{ a_{i\cdots ii\cdots i}+a_{j\cdots jj\cdots j}+r_{j}^{i}( \mathcal{A})\\ &{} + \bigl[\bigl(a_{i\cdots ii\cdots i}-a_{j\cdots jj\cdots j}-r_{j}^{i}( \mathcal {A})\bigr)^{2}+4a_{ji\cdots ii\cdots i}r_{i}(\mathcal{A}) \bigr]^{\frac{1}{2}} \bigr\} \\ \geq&U_{1}^{S}(\mathcal{A}). \end{aligned}$$

Obviously, $U_{1}^{S}(\mathcal{A})\leq\Phi(\mathcal{A})$.

(ii) Suppose that

$$\begin{aligned} U_{1}^{S}(\mathcal{A}) =& \max_{i\in S,j\in\bar{S}} \frac{1}{2} \bigl\{ a_{i\cdots ii\cdots i}+a_{j\cdots jj\cdots j}+r_{j}^{\overline{\Delta ^{S}}}( \mathcal{A}) \\ &{}+ \bigl[\bigl(a_{i\cdots ii\cdots i}-a_{j\cdots jj\cdots j}-r_{j}^{\overline {\Delta^{S}}}( \mathcal{A})\bigr)^{2}+4c_{i}(\mathcal{A})r_{j}^{\Delta^{S}}( \mathcal {A}) \bigr]^{\frac{1}{2}} \bigr\} . \end{aligned}$$

Similar to the proof of (i), we can obtain $U_{1}^{S}(\mathcal{A})\leq\Phi _{3}(\mathcal{A})\leq\Phi(\mathcal{A})$.

III. Finally, we prove that $\Psi^{S}(\mathcal{A})\leq U^{S}(\mathcal{A})$. Here, we only prove $\Psi_{1}^{S}(\mathcal{A})\leq U^{S}(\mathcal{A})$. Similarly, we can prove $\Psi_{1}^{\bar{S}}(\mathcal{A}),\Psi_{2}^{S}(\mathcal {A}),\Psi_{2}^{\bar{S}}(\mathcal{A}),\Psi_{3}^{S}(\mathcal{A}),\Psi_{3}^{\bar {S}}(\mathcal{A}),\Psi_{4}^{S}(\mathcal{A}),\Psi_{4}^{\bar{S}}(\mathcal {A})\leq U^{S}(\mathcal{A})$, respectively.

Let $i\in S$ and $j\in\bar{S}$. From the proof of Theorem 4, we can see that the bound $\Psi_{1}^{S}(\mathcal{A})$ is obtained by solving $\lambda_{0}$ from

8 $$\begin{aligned} & \bigl(\lambda_{0}-a_{i\cdots ii\cdots i}-r_{i}^{\Delta^{S}}( \mathcal{A})\bigr) \bigl(\lambda _{0} -a_{j\cdots jj\cdots j}-r_{j}^{\overline{\Delta^{S}}}( \mathcal{A})\bigr) \leq r_{i}^{\overline{\Delta^{S}}}(\mathcal{A})r_{j}^{\Delta^{S}}( \mathcal{A}). \end{aligned}$$

(i) Suppose that $r_{i}^{\overline{\Delta^{S}}}(\mathcal{A})r_{j}^{\Delta ^{S}}(\mathcal{A})=0$. If $\lambda_{0}-a_{i\cdots ii\cdots i}-r_{i}^{\Delta^{S}}(\mathcal{A})>0$, *i.e.*, $\lambda_{0}>a_{i\cdots ii\cdots i}+r_{i}^{\Delta^{S}}(\mathcal{A})$, then $\lambda_{0} -a_{j\cdots jj\cdots j}-r_{j}^{\overline{\Delta^{S}}}(\mathcal {A})\leq0$, and for any $i\in S$,

$$(\lambda_{0}-a_{i\cdots ii\cdots i}) \bigl(\lambda_{0} -a_{j\cdots jj\cdots j}-r_{j}^{\overline{\Delta^{S}}}(\mathcal{A})\bigr)\leq0\leq r_{i}(\mathcal {A})r_{j}^{\Delta^{S}}(\mathcal{A}). $$

That is to say, if $\lambda_{0}$ satisfies (8), then $\lambda _{0}$ satisfies (6), which implies that $\Psi_{1}^{S}(\mathcal{A})\leq U_{1}^{S}(\mathcal{A})\leq U^{S}(\mathcal{A})$.

If $\lambda_{0}-a_{i\cdots ii\cdots i}-r_{i}^{\Delta^{S}}(\mathcal{A})\leq0$, then $\lambda_{0} -a_{j\cdots jj\cdots j}-r_{j}^{\overline{\Delta ^{S}}}(\mathcal{A})\geq0$, *i.e.*, $\lambda_{0} \geq a_{j\cdots jj\cdots j}+r_{j}^{\overline {\Delta^{S}}}(\mathcal{A})$. From (3), we can obtain $\lambda_{0} -a_{j\cdots jj\cdots j}-r_{j}^{\overline{\Delta^{S}}}(\mathcal {A})\leq r_{j}^{\Delta^{S}}(\mathcal{A})$, *i.e.*,

9 $$\begin{aligned} & \lambda_{0} -a_{j\cdots jj\cdots j}\leq r_{j}( \mathcal{A}). \end{aligned}$$

By $\lambda_{0}-a_{i\cdots ii\cdots i}-r_{i}^{\Delta^{S}}(\mathcal{A})\leq 0\leq r_{i}^{\overline{\Delta^{S}}}(\mathcal{A})$, *i.e.*, $\lambda_{0}-a_{i\cdots ii\cdots i}\leq r_{i}(\mathcal{A})$, we have

10 $$\begin{aligned} & \lambda_{0}-a_{i\cdots ii\cdots i}-r_{i}^{\overline{\Delta^{\bar {S}}}}( \mathcal{A})\leq r_{i}^{\Delta^{\bar{S}}}(\mathcal{A}). \end{aligned}$$

Multiplying (9) with (10), we can obtain

11 $$\begin{aligned} & (\lambda_{0} -a_{j\cdots jj\cdots j}) \bigl( \lambda_{0}-a_{i\cdots ii\cdots i}-r_{i}^{\overline{\Delta^{\bar{S}}}}( \mathcal{A})\bigr)\leq r_{i}^{\Delta^{\bar {S}}}(\mathcal{A})r_{j}( \mathcal{A}), \end{aligned}$$

which implies that if $\lambda_{0}$ satisfies (8), then $\lambda_{0}$ satisfies (6), consequently, $\Psi_{1}^{S}(\mathcal{A})\leq U_{1}^{\bar{S}}(\mathcal{A})\leq U^{S}(\mathcal{A})$.

(ii) Suppose that $r_{i}^{\overline{\Delta^{S}}}(\mathcal{A})r_{j}^{\Delta ^{S}}(\mathcal{A})>0$. Then dividing (8) by $r_{i}^{\overline{\Delta^{S}}}(\mathcal {A})r_{j}^{\Delta^{S}}(\mathcal{A})$, we have

12 $$\begin{aligned} & \frac{(\lambda_{0}-a_{i\cdots i}-r_{i}^{\Delta^{S}}(\mathcal {A}))}{r_{i}^{\overline{\Delta^{S}}}(\mathcal{A})} \frac{(\lambda_{0}-a_{j\cdots j}-r_{j}^{\overline{\Delta^{S}}}(\mathcal {A}))}{r_{j}^{\Delta^{S}}(\mathcal{A})}\leq1. \end{aligned}$$

Furthermore, if $\frac{\lambda_{0}-a_{i\cdots i}-r_{i}^{\Delta^{S}}(\mathcal {A})}{r_{i}^{\overline{\Delta^{S}}}(\mathcal{A})}\geq1$, then by Lemma 2.3 in [12] and (12), we have

$$\begin{aligned} & \frac{(\lambda_{0}-a_{i\cdots i})}{r_{i}(\mathcal{A})} \frac{(\lambda_{0}-a_{j\cdots j}-r_{j}^{\overline{\Delta^{S}}}(\mathcal {A}))}{r_{j}^{\Delta^{S}}(\mathcal{A})} \leq \frac{(\lambda_{0}-a_{i\cdots i}-r_{i}^{\Delta^{S}}(\mathcal {A}))}{r_{i}^{\overline{\Delta^{S}}}(\mathcal{A})} \frac{(\lambda_{0}-a_{j\cdots j}-r_{j}^{\overline{\Delta^{S}}}(\mathcal {A}))}{r_{j}^{\Delta^{S}}(\mathcal{A})} \leq1. \end{aligned}$$

Thus, (6) holds, which implies that if $\lambda_{0}$ satisfies (8), then $\lambda_{0}$ satisfies (6), consequently, $\Psi_{1}^{S}(\mathcal{A})\leq U_{1}^{S}(\mathcal{A})$. And if $\frac{\lambda_{0}-a_{i\cdots i}-r_{i}^{\Delta^{S}}(\mathcal {A})}{r_{i}^{\overline{\Delta^{S}}}(\mathcal{A})}\leq1$, then (10) holds, which leads to (11) from (9). This implies that if $\lambda_{0}$ satisfies (8), then $\lambda_{0}$ satisfies (6), consequently, $\Psi_{1}^{S}(\mathcal{A})\leq U_{1}^{\bar{S}}(\mathcal{A})\leq U^{S}(\mathcal{A})$. The conclusion follows immediately from what we have proved. □

## Numerical examples

### Example 1

Let $\mathcal{A}=(a_{ijkl})$ be a $(2,2)$th order $3\times3$ dimensional nonnegative rectangular tensor with entries defined as follows:

$$\begin{aligned}& A(:,:,1,1)= \begin{bmatrix} 6& 1& 1\\ 1& 0& 0\\ 1& 1& 1 \end{bmatrix},\qquad A(:,:,2,1)= \begin{bmatrix} 2& 2& 0\\ 1& 1& 0\\ 2& 2& 1 \end{bmatrix},\\& A(:,:,3,1)= \begin{bmatrix} 3& 0& 3\\ 3& 2& 0\\ 1& 1& 1 \end{bmatrix}, \\& A(:,:,1,2)= \begin{bmatrix} 1& 0& 0\\ 1& 1& 1\\ 1& 2& 2 \end{bmatrix},\qquad A(:,:,2,2)= \begin{bmatrix} 2& 2& 0\\ 0& 3& 2\\ 1& 2& 0 \end{bmatrix},\\& A(:,:,3,2)= \begin{bmatrix} 1& 1& 0\\ 0& 1& 2\\ 2& 1& 2 \end{bmatrix}, \\& A(:,:,1,3)= \begin{bmatrix} 0& 1& 0\\ 1& 2& 2\\ 1& 1& 1 \end{bmatrix},\qquad A(:,:,2,3)= \begin{bmatrix} 0& 0& 1\\ 0& 0& 1\\ 0& 1& 2 \end{bmatrix},\\& A(:,:,3,3)= \begin{bmatrix} 1& 1& 1\\ 1& 0& 1\\ 2& 1& 0 \end{bmatrix}. \end{aligned}$$

By Theorem 1, we have

$$\lambda_{0}\leq33. $$

By Theorem 2, we have

$$\lambda_{0}\leq 32.8924. $$

Taking $S=\{1,2\},\bar{S}=\{3\}$, by Theorem 3, we have

$$\lambda_{0}\leq32.0540; $$

by Theorem 4, we have

$$\lambda_{0}\leq30.0965. $$

In fact, $\lambda_{0}=29.8830$. This example shows that the upper bound in Theorem 4 is smaller than those in Theorems 1-3.

### Example 2

Let $\mathcal{A}=(a_{ijkl})$ be a $(2,2)$th order $2\times2$ dimensional nonnegative rectangular tensor with entries defined as follows:

$$a_{1111}=a_{1112}=a_{1222}=a_{2112}=a_{2121}=a_{2221}=1, $$

the other $a_{ijkl}=0$. By Theorem 4, we have

$$\lambda_{0}\leq3. $$

In fact, $\lambda_{0}=3$. This example shows that the upper bound in Theorem 4 is sharp.

## Conclusions

In this paper, a new *S*-type upper bound $\Psi^{S}(\mathcal{A})$ of the largest singular value for a nonnegative rectangular tensor $\mathcal {A}$ with $m=n$ is obtained by breaking *N* into disjoint subsets *S* and its complement. It is proved that the bound $\Psi^{S}(\mathcal{A})$ is better than those in [6, 9, 10].

Note here that when $n=2$, $\Phi(\mathcal{A})=U^{S}(\mathcal{A})=\Psi ^{S}(\mathcal{A})$, and when $n\geq3$, $\Phi(\mathcal{A})\geq U^{S}(\mathcal{A})\geq\Psi ^{S}(\mathcal{A})$ always holds. How to pick *S* to make $\Psi^{S}(\mathcal{A})$ as small as possible is an interesting problem, but difficult when *n* is large. We will research this problem in the future.
